# Supplementary material for: SIRT1 Signaling Is Involved in the Vascular Improvement Induced by Moringa Oleifera Seeds during Aging
Source: Pharmaceuticals (Basel). 2023 May 18;16(5):761. doi: 10.3390/ph16050761 (PMC10221508; doi:10.3390/ph16050761)
Supplement: Supplementary file 1 [file pharmaceuticals-16-00761-s001.zip › pharmaceuticals-2375032-supplementary.pdf]

Supplementary method

Electronic paramagnetic resonance (EPR) studies for NO measurements

To assess the ability of MOI extract to induce NO release in rat vessels we measured the NO production by electron paramagnetic resonance (EPR) in rat aortic rings, exposed or not to ACh (1 μM) and/or total MOI ethanolic extract or in presence of NOS inhibitor L-NAME alone (0.1 M) as the negative control. Briefly, aortas were incubated for 45 min at 37°C in a Krebs-HEPES colloid solution containing $Fe(DETC)_2$ as a spin trap for NO detection. Then, each sample was snap-frozen in liquid nitrogen and analyzed in a Dewar flask at 77°K by an EPR Miniscope MS5000 (Frieberg Instruments, Frieberg, Germany). The instrument settings were: microwave power 10 mW; 0.400 mT amplitude modulation; 100 kHz modulation frequency; sweep time 150 s; 3 scans. Signals were quantified by measuring the total amplitude of the peaks of the spectra obtained, expressed in arbitrary units (A.U.), and normalized to the dry weight of the sample.

Data analysis

A one-way analysis of variance for repeated measures followed by a Bonferroni multiple comparisons posthoc test was used for NO measurements. Statistical significance was set at $p < 0.05$. The graphical representation of data and the statistical analyses were carried out using Stata version 16.0 (Stata Corporation, College Station, TX, USA). *$p < 0.05$, **$p < 0.01$, ***$p < 0.001$.

Supplementary results

Ethanolic extract of MOI induces NO release in the aorta

To test if the ethanolic extract of MOI was able to induce the potential release of vasorelaxant agents in conductance arteries such as the aorta, we measured NO in aortic rings using $Fe(DETC)_2$ as a spin trap and EPR. NO is the main vasorelaxant factor involved in conductance arteries harvested from YWR. As expected, NO release was increased when the aortic ring was incubated with the spin trap containing acetylcholine (ACh, 1 μM). Interestingly, the vessel could release a similar amount of NO if incubated with a solution of a spin trap containing 100 mg of the ethanolic MOI total extract (E1) (Supplementary Figure 1). If added to Ach (1 μM), E1 was unable to significantly further improve the NO release induced by ACh alone, but further increased the statistical significance compared to the control condition. Finally, the use of L-NAME (negative control) abolished also the basal level of NO released by control vessels suggesting that the signal evaluated was due to NO of NOS origin.

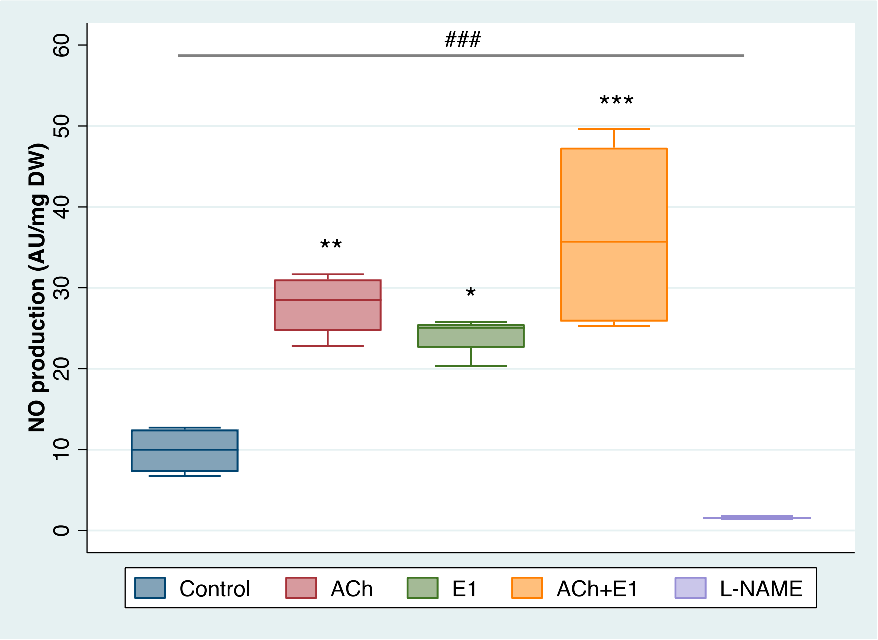

Figure S1: Evaluation of NO production by EPR. Evaluation of NO production by EPR using Fe(DETC)$_2$ as a spin trap on thoracic aortic rings with or without ACh (1 μM) or MOI ethanolic extract (100 mg/ml) alone or in association with ACh. L-NAME (100μM) condition, inhibition of NOS, was considered as the negative control. Data are expressed in Arbitrary Units (AU) and normalized to mg of Dry Weight (DW) as mean ± SEM (n=4 for each group of values), *p<0.05, **p<0.01, and ***p<0.001 versus control, ###p<0.001 versus L-NAME. One-way ANOVA analysis for repeated measures with subsequent Bonferroni's post-test. ACh, acetylcholine; E1, ethanolic MOI total extract; L-NAME, L-N^G-Nitroarginine Methyl Ester.
